# Yixinshu capsule combined with conventional treatment for chronic heart failure: Protocol for a systematic review and trial sequential analysis

**DOI:** 10.1097/MD.0000000000014960

**Published:** 2019-05-13

**Authors:** Yinhe Cai, Qingsong Zhang, Cihui Huang, Ke Lu, Baishu Chen, Cuiling Liu

**Affiliations:** a Guangzhou University of Chinese Medicine, Guangzhou; b Shenzhen Baoan Traditional Chinese Medicine Hospital Group, Shenzhen, China.

**Keywords:** chronic heart failure (CHF), meta-analysis, trial sequential analysis (TSA), Yixinshu Capsule (YXS)

## Abstract

**Background:**

Yixinshu Capsule is widely utilized in Asia for the treatment of chronic heart failure (CHF) as a conventional drug, but a comprehensive conclusion is lacking. Here, we will provide a protocol to perform a meta-analysis and trial sequential analysis to evaluate the efficacy of Yixinshu Capsule combined with conventional treatment for chronic heart failure.

**Methods:**

We will conduct a thorough search in six databases, PubMed, EMBASE, Cochrane Library Database, Chinese National Knowledge Infrastructure (CNKI), Chinese Science Journal Database (VIP), and Chinese Biomedical and Medical Database (CBM). Inclusion criteria will be randomized control trials (RCTs) with one group receiving Yixinshu Capsule based on conventional treatment and another group receiving conventional treatment alone. Modified Jadad scale and Cochrane's risk of bias assessment will be used to assess methodological quality. Only studies with modified Jadad scale score ≥3 will be included in meta-analysis for efficacy, which will be defined as moderate methodological quality. The total effective rate will be considered as the primary outcome and the secondary outcome will include mortality, rehospitalized rate, left ventricular ejection fraction (LVEF), 6-minute walking distance, E/A, left ventricular end-diastolic diameter (LVEDD), BNP, and NT-proBNP. We will conduct trial sequential analysis to evaluate the reliability of the primary outcome.

**Results:**

This study will provide a rational synthesis of current evidences for Yixinshu Capsule on chronic heart failure.

**Conclusion:**

The conclusion of this study will provide evidence for judging the efficacy of Yixinshu Capsule on chronic heart failure.

**Registration:**

PROS-PERO CRD42019119612.

## 1 Introduction

Chronic heart failure (CHF) is a life-threatening condition and usually is the final stage of many cardiovascular diseases (CVD).^[1,2]^ The prevalence of CHF is obviously severe among the quinquagenarian and the old.^[3]^ The clinical epidemiology shows the prevalence of heart failure is 0.9% in China and 20% of patients with CVD are hospitalized for CHF, with the mortality is more than 40%.^[4]^ Though abundant evidence identified that the common conventional treatment plays an important role in improving the quality of life and relieving the clinical symptoms of CHF patients, it remains far from ideal.^[5,6]^

In recent years, more and more positive evidences from clinical trials have brought growing acceptance of traditional Chinese medicine (TCM) for the treatment of cardiac diseases.^[7–9]^ Multicomponent drugs on behalf of TCM are indicating safety and efficacy in the management of heart failure.^[10,11]^ Yixinshu Capsule (YXS), a ShengMai-San-derived TCM formula, contains seven herbs, Ginseng, Salviae miltiorrhizae, Chuanxiong Rhizoma, Ophiopogonis Radix, Schisandrae Chinensis Fructus, Astragali Radix, and Crataegi Fructus, with potent effect on maintaining cardiac function by reducing oxidative stress injury and mitochondrial-mediated apoptosis.^[12,13]^ Nowadays, YXS is diffusely used in Asia, as a conventional drug, for the treatment of cardiovascular diseases.^[13,14]^ A new research, based on efficacy evaluation in vitro ad in vivo, demonstrates that Yixinshu capsule has distinctive treatment features against heart failure, which maybe particularly suitable for heart failure patients with exaltation of FABP3 and CKAP5.^[15]^ However, no interrelated meta-analysis and trial sequential analysis have been reported and no definite conclusion has been drawn on this.

Thus, we will systematically search and analyze randomized controlled trials (RCTs), with modified Jadad scale score ≥3, to evaluate the efficacy of Yixinshu Capsule combined with conventional treatment for chronic heart failure.

## 2 Methods

### 2.1 Study registration

The protocol has been registered on the International Prospective Register of Systematic Reviews (PROSPERO) with registration number CRD42019119612 basing on the Preferred Reporting Items for Systematic Reviews and Meta-Analyses Protocols (PRISMA-P) statement guidelines ^[16]^ on June 11, 2018.

### 2.2 Inclusion criteria for study selection

#### 2.2.1 Types of studies

All available randomized controlled trials (RCTs) about the use of Yixinshu capsule in patients undergoing conventional treatment for chronic heart failure will be included. Others such as retrospective study, case report, review, and studies with modified Jadad scale^[17]^ score <3 will be excluded. No restrictions were regarding languages and regions.

#### 2.2.2 Types of participants

We will include studies on patients that have been diagnosed as chronic heart failure based on New York Heart Association functional class with a history of hospitalization, with no restrictions of left ventricular ejection fraction. And patients were undergoing conventional treatment for chronic heart failure, including ARB, ACEI, β-blocker, digitalis, nitrates, diuretic, and so on. There will be no restriction on age, gender, ethnicity, and profession.

#### 2.2.3 Types of interventions

The purpose of the study is on clinical trials of Yixinshu capsule additionally based on the control group for chronic heart failure patients. Yixinshu capsule combined with other therapies will be excluded due to the efficacy of Yixinshu capsule cannot be clarified in the combined therapy. The therapeutic intervention of control group is conventional treatment for chronic heart failure.

#### 2.2.4 Types of outcome measures

##### 2.2.4.1 Primary outcome

The primary outcome is total effective rate. Total effective rate^[18]^ was primary outcome. Total effective rate = (significantly effective cases + effective cases)/number of cases. Significant effective: classification of function capacity by New York Heart Association (NYHA) increased by 2 level or above, clinical symptoms improved significantly; Effective: classification of function capacity by NYHA increased by 1 level; Ineffective: classification of function capacity by NYHA did not improve or worsen.

##### 2.2.4.2 Secondary outcomes

Secondary outcomes are mortality, rehospitalized rate, LVEF, 6-minute walking distance, E/A, LVEDD, BNP, and NT-proBNP.

### 2.3 Search methods for study identification

#### 2.3.1 Electronic searches

Relevant studies will be independently and systematically searched in PubMed, EMBASE, Cochrane Library Database, Chinese National Knowledge Infrastructure (CNKI), Chinese Scientific Journal Database (VIP), and Chinese Biomedical and Medical Database (CBM). The search items will be used as follows: Yixinshu capsule (YXS) and heart failure. Various combinations of Medical Subject Headings and non-MeSH terms will be used; the search terms with the equivalent English meaning will also be used in Chinese databases. The detailed search strategies in PubMed are provided in Table 1 and will be used similarly in other databases.

**Table 1:**
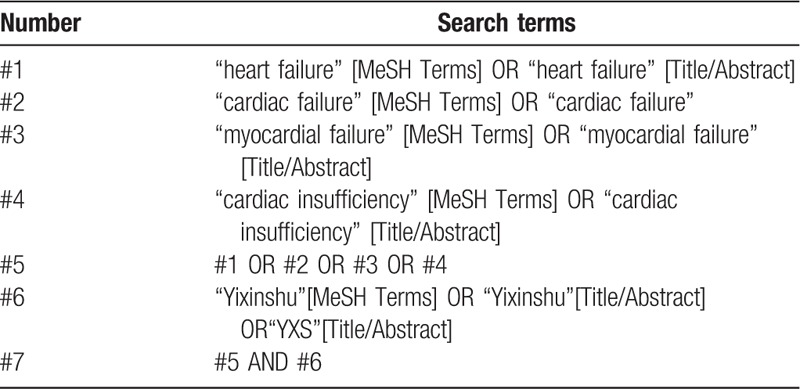
Search strategy for PubMed.

#### 2.3.2 Searching other resources

Relevant systematic review or meta-analysis of RCTs will be electronically searched. Moreover, we will filter relevant medical journals and magazines to identify literature, which is not included in the electronic databases.

### 2.4 Data collection and analysis

#### 2.4.1 Selection of studies

Two researchers will import the relevant studies obtained from the databases mentioned above into Endnote X7, a literature management software. After removing duplicates, two researchers will independently evaluate the titles and abstracts of the searched studies and exclude the significantly unqualified literature. Later, the full text of the remaining studies will be read carefully and selected according to the inclusive criteria. Any different opinions generated between the two reviewers will be resolved through discussion. When consultation fails to reach an agreement, the third reviewer will step in and provide arbitration. The study selection procedure is shown in a flow chart according to PRISMA guidelines.

#### 2.4.2 Data extraction and management

Data will be independently extracted from the included studies by two reviewers. Any divergence on data extraction will be discussed and judged by the two reviewers. The third reviewer will check the final results of the data extraction and provide arbitration for further disagreements. The following information will be extracted: author, publication year, number of participants, gender, mean age of participants, control treatment, Yixinshu capsule dose, duration, duration of follow-up, and outcomes. We will contact the corresponding author for the data mentioned above if the data are ambiguous or insufficient.

#### 2.4.3 Assessment of risk of bias in included studies

Methodological quality of untiled studies will be evaluated according to the modified Jadad quality scale^[17]^ and the Cochrane Collaboration's risk of bias tool.^[19]^ Any discrepancies in the assessment of risk of bias will be resolved by discussion and an arbiter will be consulted if it is necessary. The original Jadad scale, ranging 0 to 5 point, has been associated with an exaggeration of treatment effects, due to inadequate concealment of treatment allocation.^[20]^ We decided to use a modified Jadad scale, a 7-point system, which includes the randomization process (2 points), allocation concealment (2 points), appropriateness of blinding (2 points), and a description of dropouts and withdrawals (1 point). Studies with modified Jadad scale score ≥3 will be considered to be moderate-quality RCTs.

Cochrane Collaboration tool includes seven aspects: random sequence generation, allocation concealment, blinding of participants and investigators, blindness of outcome assessments, incomplete outcome data, selective outcome reporting, and other biases. Ultimately, the quality of the studies will be divided into three levels: “low risk of bias,” “high risk of bias,” and “unclear risk of bias.”

#### 2.4.4 Measures of treatment effect

For continuous variables, we will use weighted mean difference (WMD) to evaluate the extracted data. For dichotomous variables, rate ratio (RR) will be applied to analyze. The confidence intervals (CI) for both continuous and dichotomous variables will be set to 95%.

#### 2.4.5 Dealing with missing data

For insufficient or missing trial data, we will attempt to contact the corresponding author through e-mail if there exists any missing information, which is required. If missing data cannot be obtained, we will analyze the available data and discuss what it might cause for the review.

#### 2.4.6 Assessment of heterogeneity

We will evaluate the heterogeneity with the use of *I*^*2*^ values in accordance with the Cochrane Handbook (0%–40%, might not be important, 30%–60%, may represent moderate heterogeneity, 50%–90%, may represent substantial heterogeneity, and 75%–100% may represent considerable heterogeneity). *I*^2^ > 50% will be defined to be significant heterogeneity. The fixed-effect model will be used when there is not substantial heterogeneity; otherwise, the random-effect model will be used for meta-analysis.

#### 2.4.7 Assessment of reporting bias

Funnel plots will evaluate the presence of reporting bias for primary outcome. It will be considered that the reporting bias is existing and the reliability is low if the points on both sides of the funnel plot are dispersed and asymmetrical. Conversely, if the points on either side of the funnel plot are symmetrically distributed in substantial, we will consider the reporting bias as nonexistent and the result is reliable. And a contour-enhanced funnel plot was used to further assess for publication bias by STATA 14.0 when funnel plot was asymmetrical.^[21]^

#### 2.4.8 Data synthesis

All analyses will be conducted by using RevMan software (V5.3, The Nordic Cochrane Centre, The Cochrane Collaboration, Copenhagen, Denmark). We will select a random-effect model or fixed-effect model to merge the primary and secondary outcome indicators in accordance with the results of heterogeneity test. The fixed-effect model will be applied for data synthesis of low heterogeneity (*I*^2^ < 50%) while the random-effect model will be conducted if the heterogeneity is significant (*I*^2^ ≥ 50%). It is considered that differences are statistically significant if the results of Z test show that *P-*value is less than .05, and the 95% CI does not contain 0 (for continuous variables) or the 95% CI does not contain 1 (for dichotomous variables). A *P-*value <.05 was considered to be statistically significant.

#### 2.4.9 Subgroup analysis

We will perform a subgroup analysis to explore the potential source of the heterogeneity according to the duration for primary outcome.

#### 2.4.10 Sensitivity analysis

According to sample size, methodological quality, and the effect of missing data, we will perform sensitivity analysis to evaluate the methodological and reporting quality of the pooled studies when the outcome analyses involve a large degree of heterogeneity.

#### 2.4.11 Trial sequential analysis

TSA 9.0 version will be used to control random errors and assess imprecision. In case data are too sparse to draw firm conclusions, TSA, a sample size calculation (interim analysis), is able to widen the confidence intervals (repetitive testing).^[22]^ We will conduct TSA to investigate the reliability of the primary outcome.^[23]^ The sample size (required information size) will be calculated.

#### 2.4.12 Ethics and dissemination

Ethical approval will not be necessary because the data included in our study are derived from published literature and are not linked to individual patient data. The systematic review providing implication of the effectiveness of Yixinshu capsule combined with conventional treatment for CHF will be published in a peer-reviewed journal or conference presentations.

## 3 Discussion

Though great advances have been made in medical and device therapies for cardiovascular diseases, less improvement in outcome of general chronic heart failure population is observed.^[24]^ This situation is even worse in China, where HF prevalence was 0.9% in adults, with 1 year mortality rate of 14% and readmission rate of 41% in the latest survey of a decade ago.^[4,25]^ Though abundant evidence identified that the common conventional treatment plays an important role in improving the quality of life and relieving the clinical symptoms of CHF patients, it remains far from ideal.^[5,6]^

Yixinshu Capsule (YXS), a ShengMai-San-derived TCM formula, is widely used in Asia for the treatment of cardiovascular diseases as a conventional drug,^[13,14]^ which has a potent effect on maintaining cardiac function by reducing oxidative stress injury and mitochondrial-mediated apoptosis.^[12,13]^ And a new research^[15]^ had demonstrated that Yixinshu capsule has distinctive treatment features against heart failure, which maybe particularly suitable for heart failure patients with exaltation of FABP3 and CKAP5. However, the quality of existing randomized controlled trials (RCTs) is uneven, and there are no interrelated meta-analysis and trial sequential analysis have been reported, so no definite conclusion has been drawn on this. Therefore, the aim of this review is to collect relevant RCTs, with modified Jadad scale score ≥3, to assess efficacy of Yixinshu Capsule combined with conventional treatment for chronic heart failure.

To the best of our knowledge, it will be the first systematic review and meta-analysis on Yixinshu capsule combined with conventional treatment for CHF. First, the results of this review will provide objective statistics for further researches on Yixinshu capsule. Second, the results will offer reliable references for clinicians and patients in the treatment of CHF with Yixinshu capsule. Third, the results may introduce an alternative therapy of CHF to policy makers to decrease the burden of public health. Our aim is to provide more clinical evidence helping clinicians make decisions on clinical practice in chronic heart failure treatment.

## Author contributions

Cuiling Liu conceived the study idea. Qingsong Zhang and YinHe Cai were responsible for the design of this systematic review. CiHui Huang, Ke Lu contributed to the data analysis plan. Cuiling Liu and YinHe Cai drafted the manuscript and Baishu Chen edited. All authors provided feedback and approved the final manuscript.

**Conceptualization:** Qingsong Zhang, Cuiling Liu.

**Methodology:** Yinhe Cai.

**Project administration:** BaiShu Chen.

**Resources:** Ke Lu.

**Software:** Cihui Huang.
